# The aging ovary impairs acute stroke outcomes

**DOI:** 10.1186/s12974-023-02839-1

**Published:** 2023-07-05

**Authors:** Taylor E. Branyan, Jocelyn Aleksa, Esteban Lepe, Kelby Kosel, Farida Sohrabji

**Affiliations:** 1 Department of Neuroscience and Experimental Therapeutics, Women’s Health in Neuroscience Program, Neuroscience and Experimental Therapeutics, Texas A&M Health Science Center College of Medicine, 8447 Riverside Pkwy, Bryan, TX 77807 USA; 2 Texas A&M Institute for Neuroscience, College Station, TX 77840 USA

**Keywords:** Reproductive senescence, MCAo, Sensorimotor impairment, Ovarian cytokines, T cells, Macrophages

## Abstract

In experimental stroke, ovariectomized (OVX) adult rats have larger infarct volumes and greater sensory-motor impairment as compared to ovary-intact females and is usually interpreted to indicate that ovarian hormones are neuroprotective for stroke. Previous work from our lab shows that middle-aged, acyclic reproductively senescent (RS) females have worse stroke outcomes as compared to adult (normally cycling) females. We hypothesized that if loss of ovarian estrogen is the critical determinant of stroke outcomes, then ovary-intact middle-aged acyclic females, who have reduced levels of estradiol, should have similar stroke outcomes as age-matched OVX. Instead, the data demonstrated that OVX RS animals showed better sensory-motor function after stroke and reduced infarct volume as compared to ovary-intact females. Inflammatory cytokines were decreased in the aging ovary after stroke as compared to non-stroke shams, which led to the hypothesis that immune cells may be extravasated from the ovaries post-stroke. Flow cytometry indicated reduced overall T cell populations in the aging ovary after middle cerebral artery occlusion (MCAo), with a paradoxical increase in regulatory T cells (Tregs) and M2-like macrophages. Moreover, in the brain, OVX RS animals showed increased Tregs, increased M2-like macrophages, and increased MHC II + cells as compared to intact RS animals, which have all been shown to be correlated with better prognosis after stroke. Depletion of ovary-resident immune cells after stroke suggests that there may be an exaggerated response to ischemia and possible increased burden of the inflammatory response via extravasation of these cells into circulation. Increased anti-inflammatory cells in the brain of OVX RS animals further supports this hypothesis. These data suggest that stroke severity in aging females may be exacerbated by the aging ovary and underscore the need to assess immunological changes in this organ after stroke.

**Supplementary Information:**

The online version contains supplementary material available at 10.1186/s12974-023-02839-1.

## Background

In the United States, stroke is a leading cause of death and long-term disability. Women tend to suffer worse stroke, with greater rates of disability and mortality, than men of the same age [1]. Stroke risk in women is modified across their lifespan. In premenopausal age groups, women have a lower risk of stroke; however, around perimenopause, female stroke risk meets and then later exceeds that of men [2]. This is largely thought to be due to the loss of neuroprotective ovarian hormones, such as estrogens, though the Women’s Health Initiative demonstrated that hormone replacement therapy is not a viable stroke preventative in older women, as women in the group who received hormone replacement therapy experienced more strokes and more stroke-related mortality than women in the placebo group [3]. This finding is also supported with preclinical evidence from several labs, which shows that ovariectomizing young, normally cycling mature adult (3 mos, or 5-7mos) female mice and rats drastically increases infarct volume and worsens sensorimotor deficit though estrogen replacement rescues this effect [4–6]. However, ovariectomy with estrogen replacement to reproductively senescent (9-11mos, acyclic) female rats (referred to in this paper as RS) results in much worse stroke outcomes compared to the OVX reproductively senescent female [7, 8]. Though the effects of estrogens have been widely studied in the context of stroke and sex differences, the role of the ovary beyond just hormone production has been largely ignored.

The ovary serves as home to a large population of immune cells that play a role in normal ovarian function, such as menstruation, ovulation, and reproduction. For example, regulatory T cells (Tregs) are necessary to prevent a destructive immune response to conception [9] and likely prevent spontaneous abortion [10]. Moreover, Treg deficiency disrupts ovarian steroid production and results in premature ovarian insufficiency, a condition where ovarian function is compromised before age 40 and is linked to increased cardiovascular risk [11]. Impaired function of both CD4 + and CD8 + T cells and abnormal cytokine production is also associated with polycystic ovarian syndrome (PCOS) [12, 13]. Macrophage pyroptosis, a highly inflammatory form of lytic cell death, in the ovaries is also associated with the development of PCOS and eliminating this specific cell death pathway is sufficient to ameliorate PCOS symptoms in mice [14]. Like premature ovarian insufficiency, PCOS is linked to increased risk for adverse cardiovascular events (reviewed in [15]). Despite these links to aberrant ovarian function and increased cardiovascular risk, no studies have directly assessed how a stroke or other adverse cardiovascular event may affect the ovary or how the health of the ovary, beyond hormone production, may influence stroke outcomes. Therefore, this study is the first to assess how the aging ovary responds to and impacts acute stroke outcomes.

## Methods

### Animals

All animals were purchased from Envigo (IN). Animals were purchased as adult proven breeders (5–7 months, 230–320 g) or middle-aged retired breeders (9–11 months, 280–360 g). All animals were maintained in a 12-h dark:12-h light cycle in AAALAC-accredited vivarium facilities. Food and water were available ad libitum. A week after arrival, females were assessed for vaginal cytology daily for 14 days to determine estrus status [16]. Vaginal cells were collected using cotton swabs, placed on slides and cytology was examined at a low magnification. Adult females with a normal estrous cycle of 4–6 days were included in the study. Middle-aged females were included if cytology indicated they were in constant diestrus for at least 7 consecutive days. Adult animals and middle-aged animals were at an average of 7 and 11.5 months, respectively, at the time of middle cerebral artery occlusion (MCAo). Within each age, animals were randomly assigned randomly to groups. Animals were anesthetized for all surgical procedures with xylazine (200 mg/kg)/ketamine (10 mg/kg). A total of 155 animals were used in these studies with group sizes of 5–14. All experimental procedures were conducted in accordance with ARRIVE guidelines [17].

### Blood draws

Blood from the saphenous vein was collected using a 27-gauge needle to puncture the vein and then collected in an Eppendorf tube. The blood was spun down at 2500 × g for 15 min at 4 °C. Blood draws were performed 2 days prior to OVX (PRE OVX), 2 days post OVX (POST OVX), 2 prior to MCAo (PRE MCAo), and 2 days post-MCAo (2DP).

### Ovariectomy (OVX)

After determining estrus cyclicity or acyclicity for each age group, animals either underwent bilateral ovariectomy or sham ovariectomy surgery. Ovaries were removed by making a dorsal midline incision inferior to the rib cage and kidneys according to our previously established procedures [7, 18]. Ovaries were flash frozen for later analysis. A subset of animals received a sham ovariectomy surgery (referred to as ovary intact), in which the same incisions were made, but the ovaries were not removed. Animals were allowed to recover for 3 weeks before MCAo surgery. Body weights were taken prior to OVX, at the time of stroke surgery, and at the time of termination.

### Middle cerebral artery occlusion (MCAo)

All animals were subjected to stereotaxic surgery to occlude the left middle cerebral artery as reported in [19–21]. Briefly, MCA occlusion was induced by stereotaxic microinjection of Endothelin-1 (1.5 μg in 3 μl of Dulbecco’s Phosphate Buffered Saline, Bachem, CA). ET-1 was injected adjacent to the middle cerebral artery at the following coordinates relative to bregma: + 0.9 mm anterior/posterior, + 3.4 medial/lateral, and − 8.5 relative to the dura. Sensorimotor tests were performed 2 days prior to MCAo as well as 2 and 5 days post-MCAo to assess the stroke-induced deficit. Animals were terminated at 5 days post-stroke, and survival was monitored throughout.

### Infarct volume

Infarct volume estimation was performed on animals terminated on day 5 post-stroke using our previously described procedures[7]. Briefly, brain slices (2 mm thick) between − 2.00 mm and + 4.00 mm from Bregma were incubated in a 2% triphenyl tetrazolium chloride solution at 37 °C for 20 min and then photographed using a Nikon E950 digital camera attached to a dissecting microscope. Digitized images were coded and analyzed by an investigator blind to the code. Infarct volume was determined using ImageJ (NIH) and normalized to the volume of the contralateral hemisphere.

### Behavioral assays

Adhesive removal task: sensory-motor performance was assessed using procedures described previously for the adhesive-removal test [22, 23]. Briefly, two pieces of adhesive-backed foam tape (1 X ½”) were attached to the palmar surface of the paw of each forelimb. For each forelimb, the time it took to remove the tape from the forelimbs was recorded during three trials per day. Animals were allowed to rest for 5 min between sessions, and each test session had a maximum time limit of 120 s. Vibrissae-evoked forelimb placement task: stroke injury was assessed using the vibrissae-evoked forelimb placement task, which was performed pre- and post-MCAo (described by [7, 24]). Briefly, animals were subjected to same-side placing trials and cross-midline placing trials by stimulating ipsilesional and contralesional vibrissae to elicit a forelimb placing response.

### Protein extraction

Tissue from the ischemic hemisphere (cortex and striatum) or from ovaries from animals terminated at 48 h were harvested and lysed in RIPA lysis buffer (Thermo Scientific, Grand Island, NY) and centrifuged at 15,000 rpm for 15 min. Supernatant was collected and stored at -20 °C until further analysis. Protein concentrations were determined using the BCA protein assay kit (Pierce, Rockford, IL).

### Multiplex cytokine ELISA

Cytokine levels from serum, ovarian homogenate, and brain homogenate were measured using a multiplexed rat chemokine/cytokine panel which detects 27 analytes (Millipore, MA). The ELISAs were performed according to manufacturer’s directions and our previously described protocols [25, 26].

### Steroid analysis

Serum and ovary samples were sent to the Wisconsin National Primate Center for steroid analysis using LC–MS/MS for 6 analytes (testosterone, progesterone, estrone, estradiol, androstenedione, and 17-OH Progesterone) using a 6500 Mass Spectrometer.

### Flow cytometry

The procedure for cell isolation for flow cytometry was adapted from [27]. Briefly, ovaries and lymph nodes (cervical, para-aortic, mesenteric) were collected from female rats 2 days post-MCAo and roughly chopped and digested in a buffer containing Collagenase II (1 mg/ml), DNAase I (0.15 mg/ml), and Dispase II (1 mg/ml) using the gentle MACS™ Octo dissociator for 30 min at 37ºC. The ischemic hemisphere was also removed, roughly chopped, and digested using the Adult Brain Dissociation Kit (Miltenyi) using the gentle MACS™ Octo dissociator for 30 min at 37 ºC. Cells were then washed with cold DPBS and filtered through a 70-um cell strainer. For brain samples, a myelin debris removal step was performed. Red blood cells were lysed by incubating cells with ACK lysing buffer for 3 min at room temperature. The Fc component of antibodies was blocked by using an anti-CD16/32 antibody (BD Bioscience) and stained with a live/dead antibody (Ghost Dye™ Red 780 Viability Dye, Cell Signaling Technologies). Cells were then split into two samples and stained with either the T cell panel or the macrophage panel. T cell panel: CD45-FITC (Clone OX-1, BD Biosciences), CD3-BV421 (Clone 1F4, BD Biosciences), CD4-BV605 (Clone OX-35, BD Biosciences), CD8a-PE Vio770 (Clone REA437, Miltenyi Biotec), TCR $\gamma \delta$-PE (Clone V65, BioLegend), FoxP3-APC (Clone FJK-16 s, Thermo Fisher Scientific), and CD25-BV750 (Clone OX-39, BD Biosciences). Macrophage panel: CD45-FITC (Clone OX-1, BD Biosciences), CD68-PE Vio770 (Clone REA237, Miltenyi Biotec), Csf1R/CD115-PE (Clone 604B5 2E11, Thermo Scientific), CD80-BV605 (Clone B7-1, Fisher Scientific), and MHCII-PerCP Fluor710 (Clone OX-17, Thermo Scientific). Samples were run on a BD Fortessa X-20 cytometer. Results are expressed as percentage of CD45 + cells. Unstained samples and compensation controls were used to set the gating strategy. Fluorescence minus one (FMO) was used when necessary to identify and gate positive populations. Data were shown as either pseudocolor or contour plots based on best visualization of the data.

### Statistical analysis

Statistical analysis was performed using GraphPad Prism Version 9.4.1. Comparisons were made using either a two-tailed Student’s t-test or one- or two-way ANOVAs. Pearson’s correlation table was generated using inputs from ovarian and serum cytokine expression derived from multi-plexed ELISA assays and from 2-day ART performance.

Significance level for all tests was set at *p* $\le$ 0.05, and all data are expressed as mean $\pm$ SEM. Specific *p*-values, statistical test used, and sample size are reported in figure legends. The Metaboanalyst program (https://www.metaboanalyst.ca) was used for dimensionality reduction tools (sparse Partial Least Squares Discriminant Analysis [sPLS-DA], Heatmaps).

## Results

### Ovariectomy of RS animals improves acute stroke outcomes

**Fig. 1:**
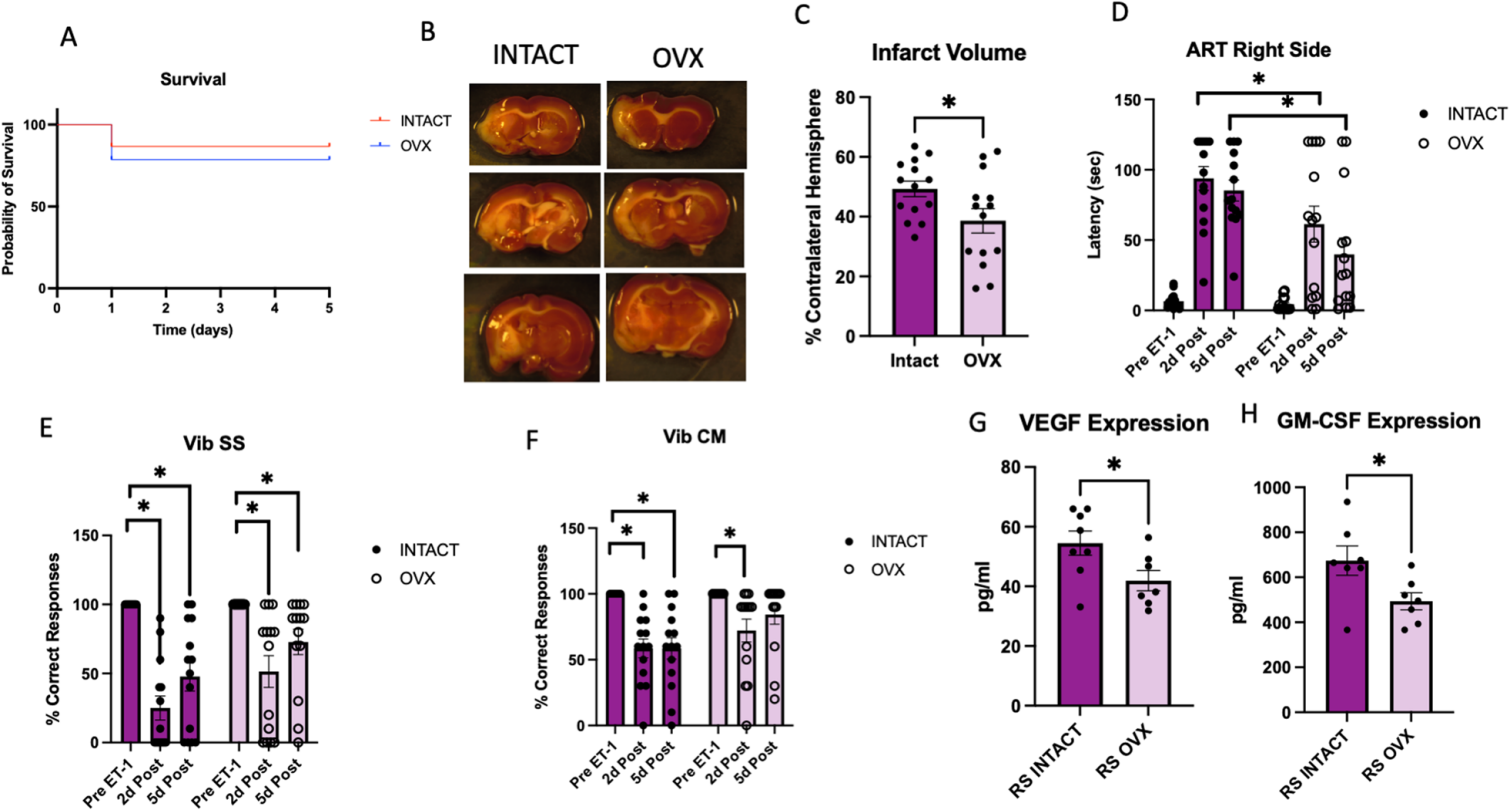
Effect of ovariectomy on acute stroke outcomes in RS females. **A** Kaplan–Meier survival plot. **B** Representative images of TTC-stained sections obtained 5d post-MCAo. **C** Histogram depicting mean (± SEM) infarct volume. **D** Histogram of mean (± SEM) latency to remove the adhesive tape pre-, 2 days post-, and 5 days post-MCAo. **E** Vibrissae-evoked forelimb placement task for same-side stimulation. **F** Vibrissae-evoked forelimb placement task for cross-midline stimulation. **G** Expression of VEGF and (H) GM-CSF in the ischemic hemisphere. *N* = 14 (intact) and n = 14 (OVX) for **A**–**F**. *N* = 8 (intact) and *n* = 7 (OVX) for **G**, **H**. *p ≤ 0.05

To assess the contribution of the aging ovary acute stroke outcomes, a bilateral ovariectomy (OVX) or sham ovariectomy (Intact) was performed on anesthetized female RS rats. OVX resulted in significantly increased body weight at the time of stroke, and this increase persisted to the time of termination although percent weight loss after stroke was not different in intact and OVX groups (Additional file 1: Fig. S1A, B). There were no differences in survival between the OVX and intact groups (Fig. 1A); however, there was a significant decrease in infarct volume in the OVX group compared to the intact group (Fig. 1B, C). There was no difference in spleen weights between both groups (Additional file 1: Fig. S1C). A stroke effect was observed in performance on sensorimotor tasks, as animals in both groups showed significantly greater latency in ART post-stroke. However, the OVX group demonstrated significantly reduced latency compared to the intact group, indicating less severe deficits were sustained (Fig. 1D). A stroke effect was seen in both groups on the same-side VIB task, with no OVX effect (Fig. 1E). The cross-midline VIB task showed a deficit in performance at both 2 and 5 days post-stroke in the intact group, while the OVX group demonstrated a deficit at 2 days post-stroke that recovered at 5 days post-stroke (Fig. 1F). A multiplexed cytokine/chemokine ELISA was performed on protein isolated from the ischemic hemisphere of these animals, and only 2 analytes were regulated by OVX. Specifically, both VEGF and GM-CSF were significantly reduced in the OVX group as compared to the intact group (Fig. 1G, H).

To determine the effect of OVX on circulating inflammatory cytokines after stroke, cytokine analysis of serum from Adult and RS animals pre-OVX and 2 days post-stroke was performed. For EGF, there was an effect of stroke, an effect of OVX, and an interaction effect between stroke and OVX, with a significant increase in EGF expression after stroke in the RS OVX group (Additional file 1: Fig. S2A). For MCP-1, there was an effect of age and an interaction effect between age and OVX, with a significant difference in expression between the Adult Intact and RS Intact groups (Additional file 1: Fig. S2B). MIP-2 showed a stroke effect and an interaction effect between stroke, age and OVX, with a significant decrease in the RS OVX group post-stroke (Additional file 1: Fig. S2C). Finally, IL-1 $\alpha$ shows a stroke effect with a significant decrease in the RS OVX group post-stroke (Additional file 1: Fig. S2D). This analysis suggests that the RS OVX group may have a lower circulating inflammatory profile after stroke compared to their age-matched intact counterparts.

### Stroke significantly reduced ovarian steroid hormone expression in adult animals with muted effects in RS animals

**Fig. 2:**
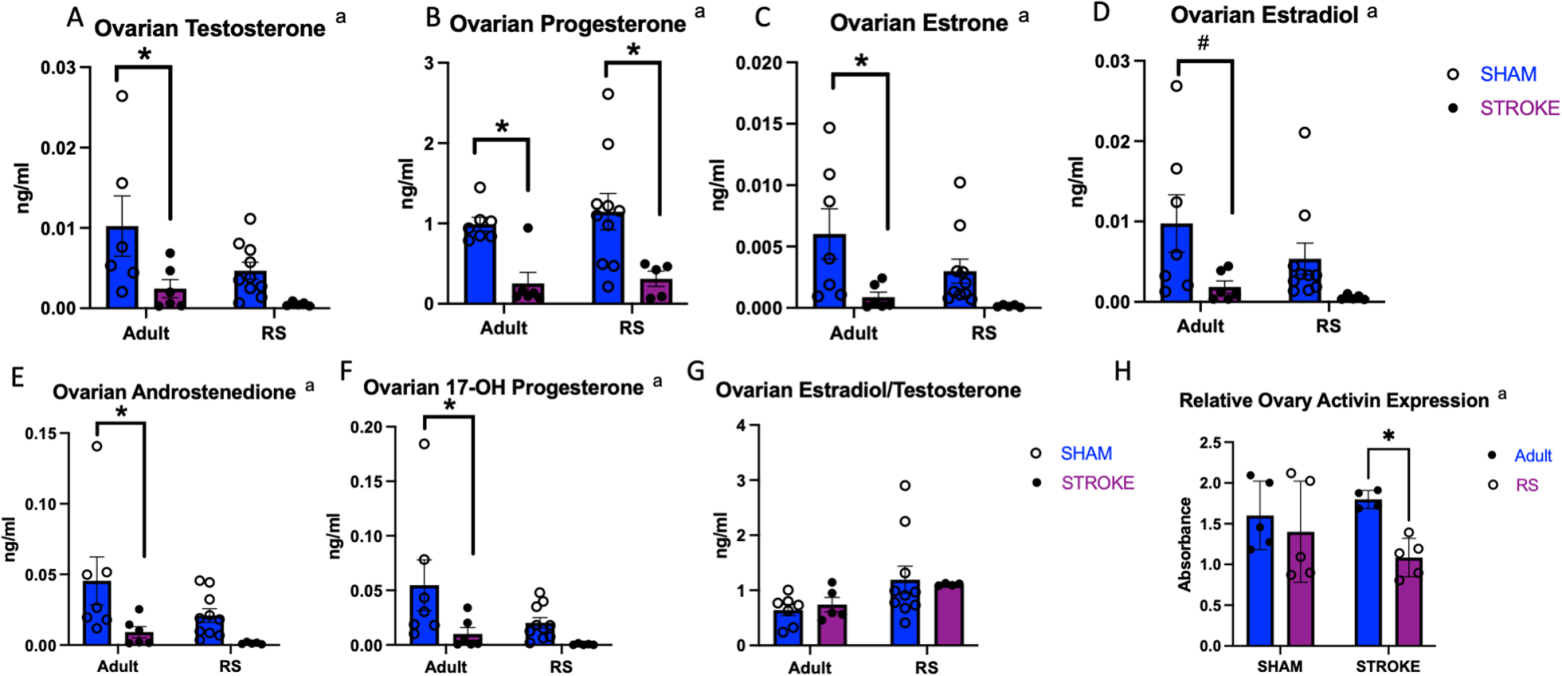
Effect of stroke on ovarian steroid hormone expression in adults and RS animals. LC–MS analysis of testosterone (**A**), progesterone (**B**), estrone (**C**), estradiol (**D**), androstenedione (**E**), 17-OH progesterone (**F**) and the ratio between estradiol and testosterone (**G**) in Adult and RS sham and stroke ovaries. **H** ELISA analysis of activin in Adult and RS sham and stroke ovaries. All samples were collected 2 days post-stroke, and a two-way ANOVA for age and stroke condition was used to compare groups. *N* = 7 Adult stroke, *N* = 6 Adult sham, *N* = 10 RS sham, *N* = 5 RS stroke. **a** Effect of stroke, **b** effect of age, **c** interaction effect. *p ≤ 0.05

To assess the effect of stroke on ovarian and circulating gonadal hormone production, ovaries and serum from RS and adult control and stroke animals were sent to the Wisconsin National Primate Center for mass spectrometry analysis. In the Adult ovaries, stroke resulted in a significant decrease in testosterone, progesterone, estrone, estradiol, androstenedione, and 17-OH progesterone (Fig. 2A–F). The RS ovaries showed a decrease in progesterone after stroke (Fig. 2B), but there was no significant difference in any other hormone expression with regard to stroke. Stroke did not affect the ratio of estradiol to testosterone in the ovaries (Fig. 2G). Overall, expression of the hormones trended lower in the RS ovaries as compared to the Adult ovaries. Serum analysis of these hormones indicated that OVX significantly affects circulating estrone levels (Additional file 1: Fig. S3A) and that ratio of circulating estradiol to testosterone was significantly decreased in the RS OVX group as compared to the RS intact group (Additional file 1: Fig. S3B–D). ELISA analysis of other peptide hormones indicated that the RS ovary shows reduced activin expression as compared to the Adult ovary post-stroke (Fig. 2H).

### Stroke reduced inflammatory cytokine expression in RS ovaries

**Fig. 3:**
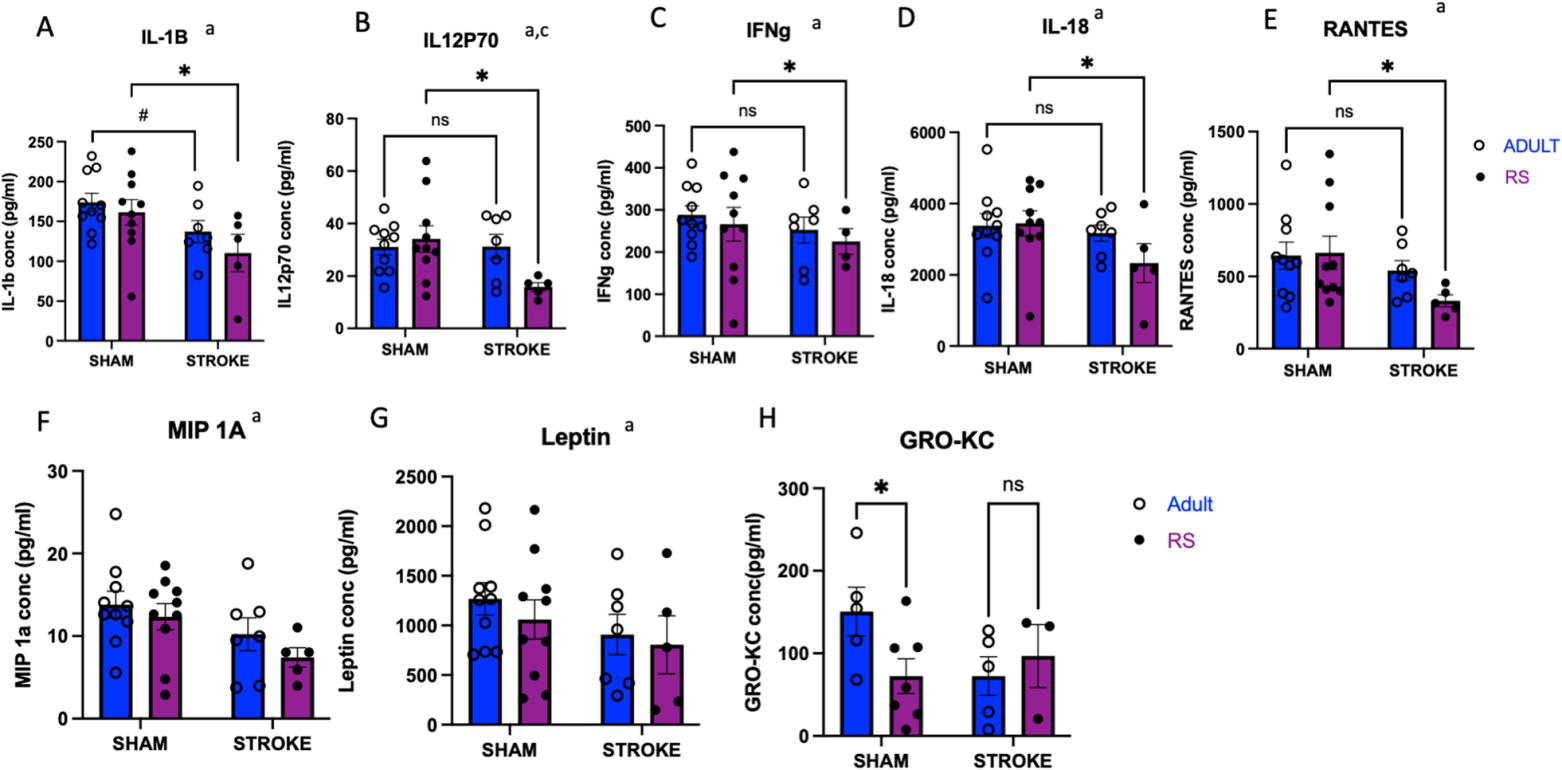
Effect of stroke on inflammatory cytokine production in the ovary. Multiplexed cytokine analysis of IL-1 $\beta$ (**A**), IL12p70 (**B**), IFN $\gamma$ (**C**), IL-18 (**D**), Rantes (**E**), MIP1-$\alpha$ (**F**), Leptin (**G**), and Gro-KC (**H**) of Adult and RS sham and stroke ovaries. All samples were collected 2 days post-stroke, and a two-way ANOVA for age and stroke condition was used to compare groups. *N* = 10 Adult sham, *N* = 10 RS sham, *N* = 7 Adult stroke, *N* = 5 RS stroke. Main effect of stroke; **b** main effect of age; **c** interaction of stroke by age. *p ≤ 0.05

Though differences in hormone expression were observed in RS versus adult ovary, the differences were modest, we next assessed how the aging ovary affects inflammation post-stroke. A multiplexed chemokine/cytokine ELISA was performed on homogenized ovarian tissue from Adult and RS rats 2 days after stroke or sham surgery. IL-1 $\beta$, IL-12p70, IFN $\gamma$, IL-18, RANTES, MIP-1 $\alpha$, and Leptin demonstrate a significant effect of stroke (Fig. 3A–G), indicating that the ovary does alter its cytokine production in response to stroke. Moreover, IL-1 $\beta$, IL-12p70, IFN $\gamma$, IL-18, and RANTES all exhibit a significant decrease in ovarian expression between the RS Sham and RS Stroke groups (Fig. 3A–E), whereas there is no such difference in the Adult groups, though there is a trending decrease in IL-1 $\beta$ between the Adult Sham and Adult Stroke groups (Fig. 3A). The decrease in cytokine expression in the aging ovaries post-stroke may be indicative of extravasation of the immune cells post-stroke, which is a well-accepted phenomenon that occurs in other secondary lymphoid organs, such as the gut [28]. Additionally, there was a significant reduction GRO-KC expression in the RS Sham ovary as compared to the Adult Sham ovary, though there was no effect of stroke for this cytokine (Fig. 2H).

**Fig. 4:**
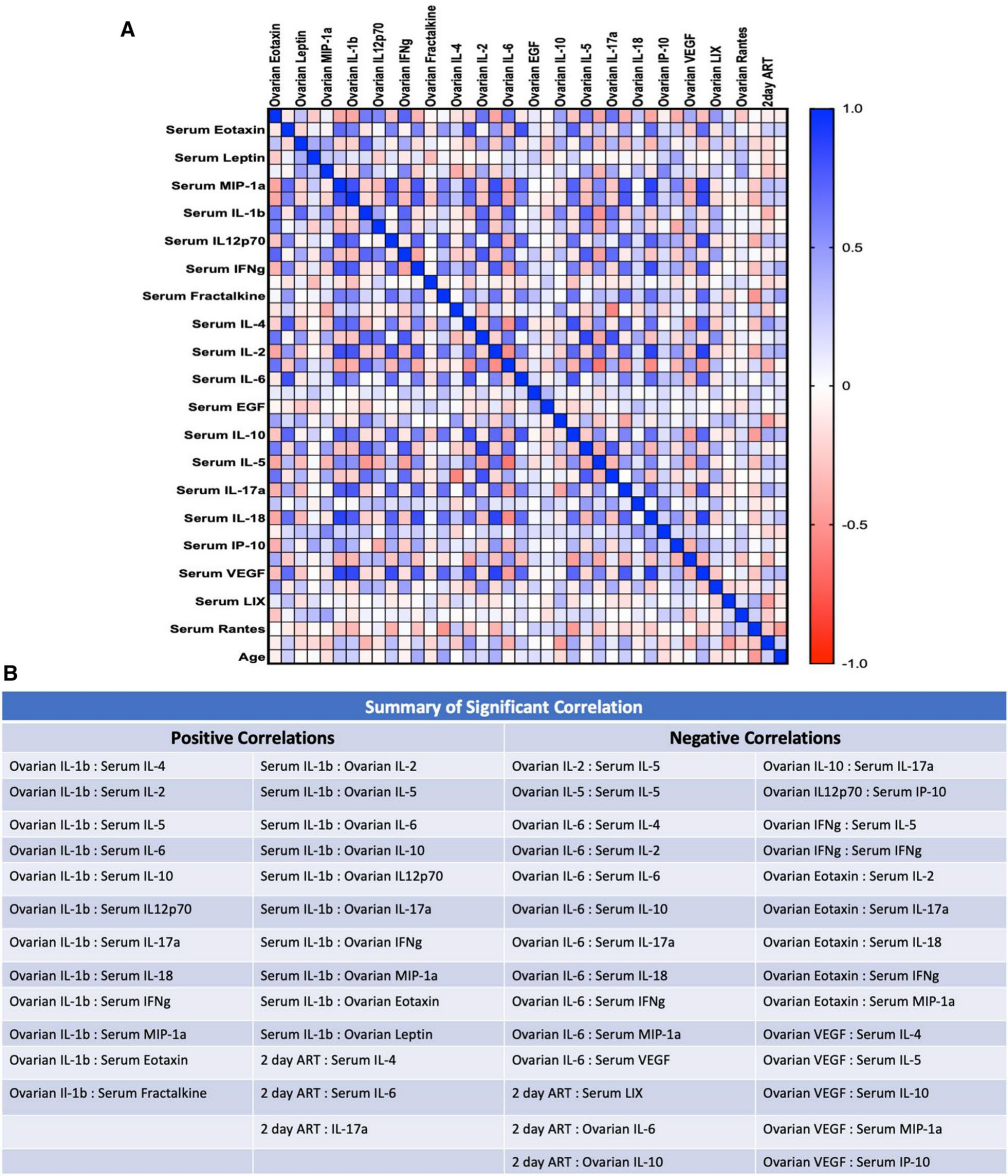
Ovarian–serum cytokine correlation matrix. **A** Pearson’s correlation matrix visualizing correlations between ovarian and serum cytokine expression. **B** Summary of significant correlations for **A**

For comparison, sera from the same animals used for the ovarian cytokine analysis were also subjected to a multiplex ELISA. IL-1 $\beta$, IL-17 $\alpha$*,* Eotaxin, MCP-1, and IL-4 all exhibit a significant increase in the Adult Stroke group as compared to the Adult Sham group, whereas this increase is not seen in between the RS Stroke and RS Sham groups (Additional file 1: Fig. S4A–E). Though not significant, expression of these cytokines trended higher in the RS Sham group as compared to the Adult Sham group, suggesting that baseline inflammation may be higher in the older females. Additional evidence of this ‘inflammaging’ in the RS animals is demonstrated by the fact that MCP-1, G-CSF, TNF-$\alpha ,$ fracktalkine, IL-5, IL-12p70, RANTES, and IL-2 show an effect of age, with the RS animals exhibiting greater expression of these cytokines (Additional file 1: Fig. S4E–L). To assess how ovarian cytokine expression may influence circulating cytokine expression, a Pearson’s correlation matrix was created using expression values from the two multiplex ELISAs previously described, shown in Fig. 4A, with significant correlations summarized in Fig. 4B. With the exception of IL-1 $\beta$, significant correlations between ovarian and serum cytokines primarily had a negative R value, indicating that there is an inverse relationship between expression of these cytokines in the ovary versus in the serum. Moreover, there were significant negative correlations between ovarian IL-6 and ovarian IL-10 expression and 2d ART performance, suggesting that ovarian cytokine expression may directly affect stroke outcomes.

### Stroke causes an overall depletion of T cells in the aging ovary yet retains Tregs and M2-like macrophages

**Fig. 5:**
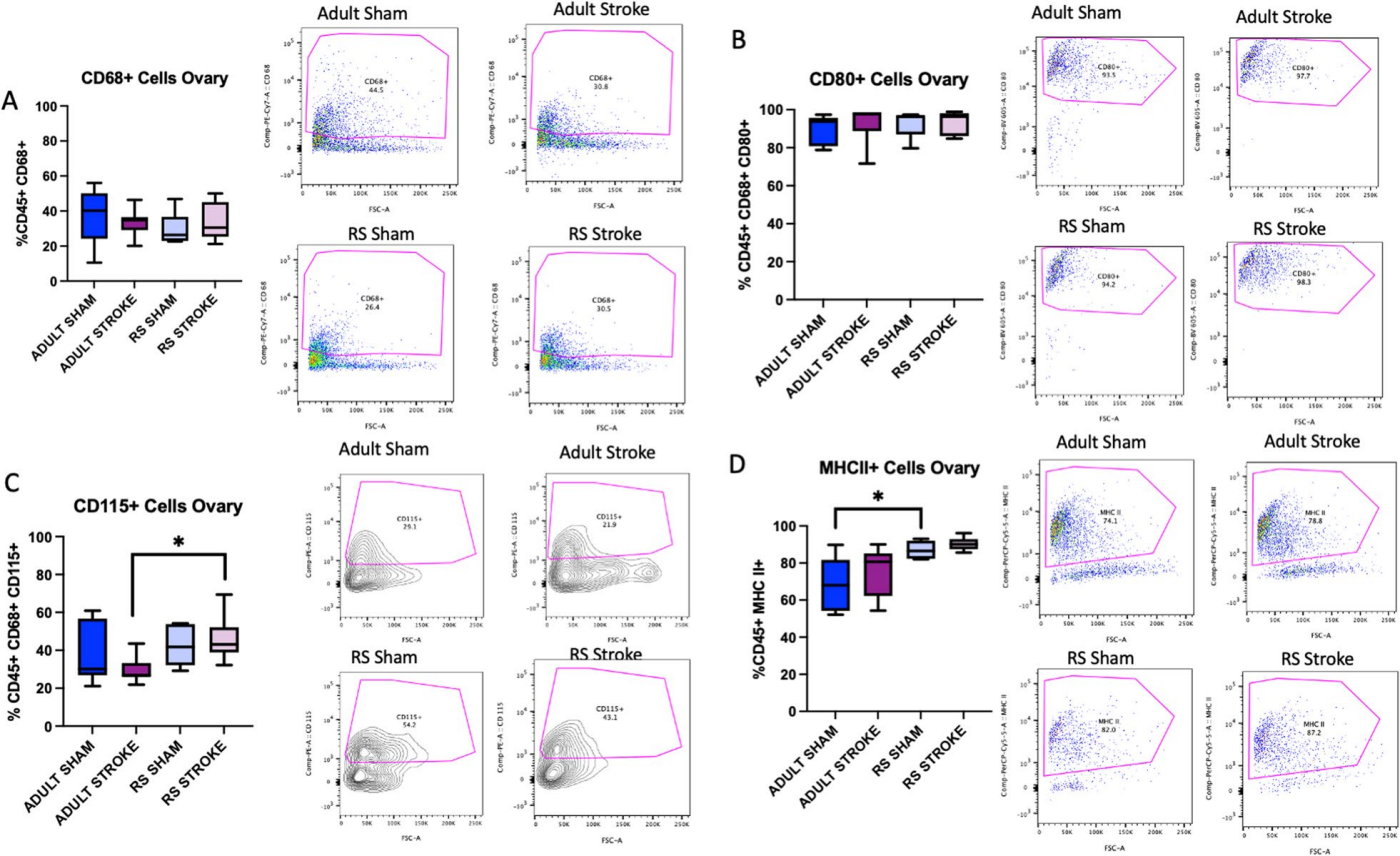
Macrophage flow cytometry analysis of ovaries. Flow cytometry analysis of CD68 + (**A**), CD80 + (**B**), CD115 + (**C**), and MHCII + (**D**) cells in the Adult and RS sham and stroke ovary. All samples were collected 2 days post-stroke, and a two-way ANOVA for age and stroke condition was used to compare groups. *N* = 6 Adult sham, *N* = 8 Adult stroke, *N* = 5 RS sham, *N* = 8 RS stroke. *p ≤ 0.05

**Fig. 6:**
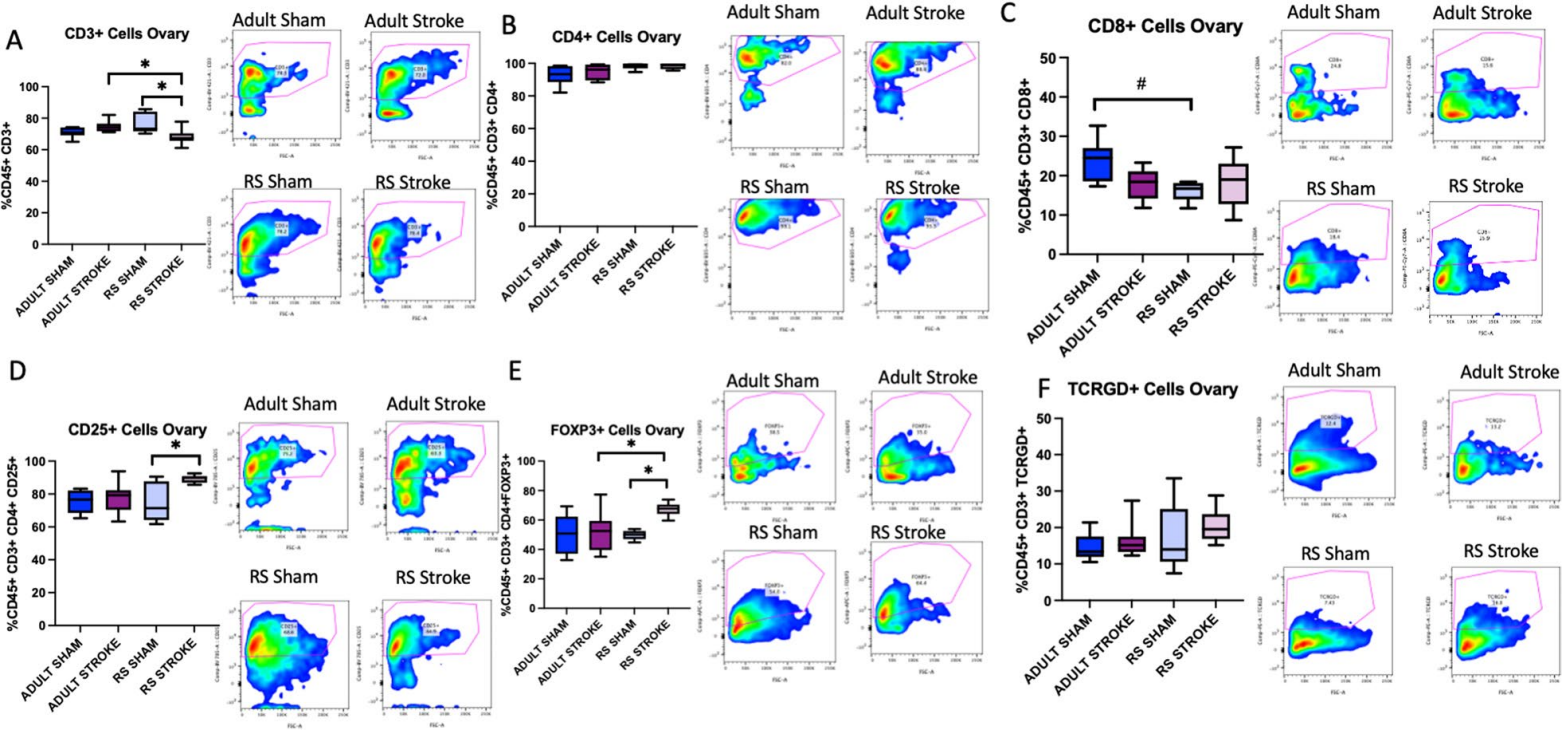
T cell flow cytometry analysis of ovaries. Flow cytometry analysis of CD3 + (**A**), CD4 + (**B**), CD8 + (**C**), CD25 + (**D**), FoxP3 + (**E**), and TCRGD + (**F**) cells in the Adult and RS sham and stroke ovary. All samples were collected 2 days post-stroke, and a two-way ANOVA for age and stroke condition was used to compare groups. *N* = 5 Adult sham, *N* = 7 Adult stroke, *N* = 5 RS sham, *N* = 9 RS stroke. *p ≤ 0.05

Based on the cytokine data, we hypothesized that stroke may cause immune cells to be extravasated from the ovaries. To test this hypothesis, ovaries were collected 2 days post-sham or post-stroke surgery, processed into a single cell suspension, stained with antibodies from either a macrophage or T cell panel, and counted on a cytometer. This acute timepoint was chosen to assess ovarian immune function as immune cell infiltration into the brain is known to occur very early after stroke, peaking around 2 days post-stroke for neutrophils and T cells [29]. The gating strategy is outlined in Additional file 1: Fig. S5A–D. There was no difference in the proportion of macrophages (CD68 + cells) in the Adult or RS ovary after stroke (Fig. 5A). To assess macrophage phenotype, CD80 was chosen as a M1 macrophage marker and CD115 was chosen as an M2 macrophage marker, though there was significant co-staining of these two antibodies in the ovaries, suggesting that the macrophages in the ovary likely exist on a spectrum of reactivity rather than being completely polarized to an M1 or M2 phenotype. No differences were observed in the populations of CD80 + macrophages within the ovary (Fig. 5B); however, the proportion of CD115 + cells in the Adult stroke ovary was significantly lower than the proportion of CD115 + cells in the RS ovary (Fig. 5C, *p*= 0.0443). Also, there was significantly more MHCII + cells in the RS sham ovary as compared to the Adult sham ovary (Fig. 5D, *p*= 0.0292). There was also decrease in CD3 + cells in the RS ovaries post-stroke as compared to sham (Fig. 6A, *p* = 0.0141), and this decrease was not observed in the Adult ovaries. There were no differences in CD4 + or in CD8 + T cells in the ovaries after stroke (Fig. 6B, C), though the overall percentage of CD4 + T cells in all the ovary groups was very high. However, there was a trend for fewer CD8 + T cells in the RS Sham ovaries as compared to the Adult Sham ovaries (Fig. 6C, *p*= 0.0700). There was a significant increase in CD25 + T cells in the RS ovary post-stroke (p = 0.0227) with no change in this population in the Adult ovary (Fig. 6D), and the proportion of FoxP3 + cells in the RS stroke ovary was significantly different from the Adult stroke (p = 0.0297) and the RS sham groups (Fig. 6E, *p*= 0.0229). The proportion of $\gamma \delta$ T cells was not altered in any condition (Fig. 6F). Overall, these data indicate that the immune cells populations in the ovary change with stroke, and that the aging ovary may deplete more inflammatory immune cells, as indicated by the decrease in overall T cells, while retaining anti-inflammatory immune cells, such as CD25 + and FoxP3 + T cells and CD115 + macrophages.

### Ovariectomy increases M2-like macrophages, MHC II + cells, and Tregs in the brain post-stroke

To assess how the aging ovary affects neural immune cell populations post-stroke, RS females were ovariectomized, allowed to recover for three weeks, subjected to MCAo, and then terminated at 2 days post-stroke. The ischemic hemisphere as well as cervical, para-aortic, and mesenteric lymph nodes were collected and processed for flow cytometry. Cervical lymph were selected due to proximity to the brain, para-aortic lymph nodes were selected because the ovaries drain to these nodes, and the mesenteric lymph nodes were selected due to the proximity to the gut, which is a secondary lymphoid organ that has been shown to traffic immune cells to the brain post-stroke [30]. The same macrophage and T cell panels that were used for the ovaries were used to stain the brain and lymph nodes.

**Fig. 7:**
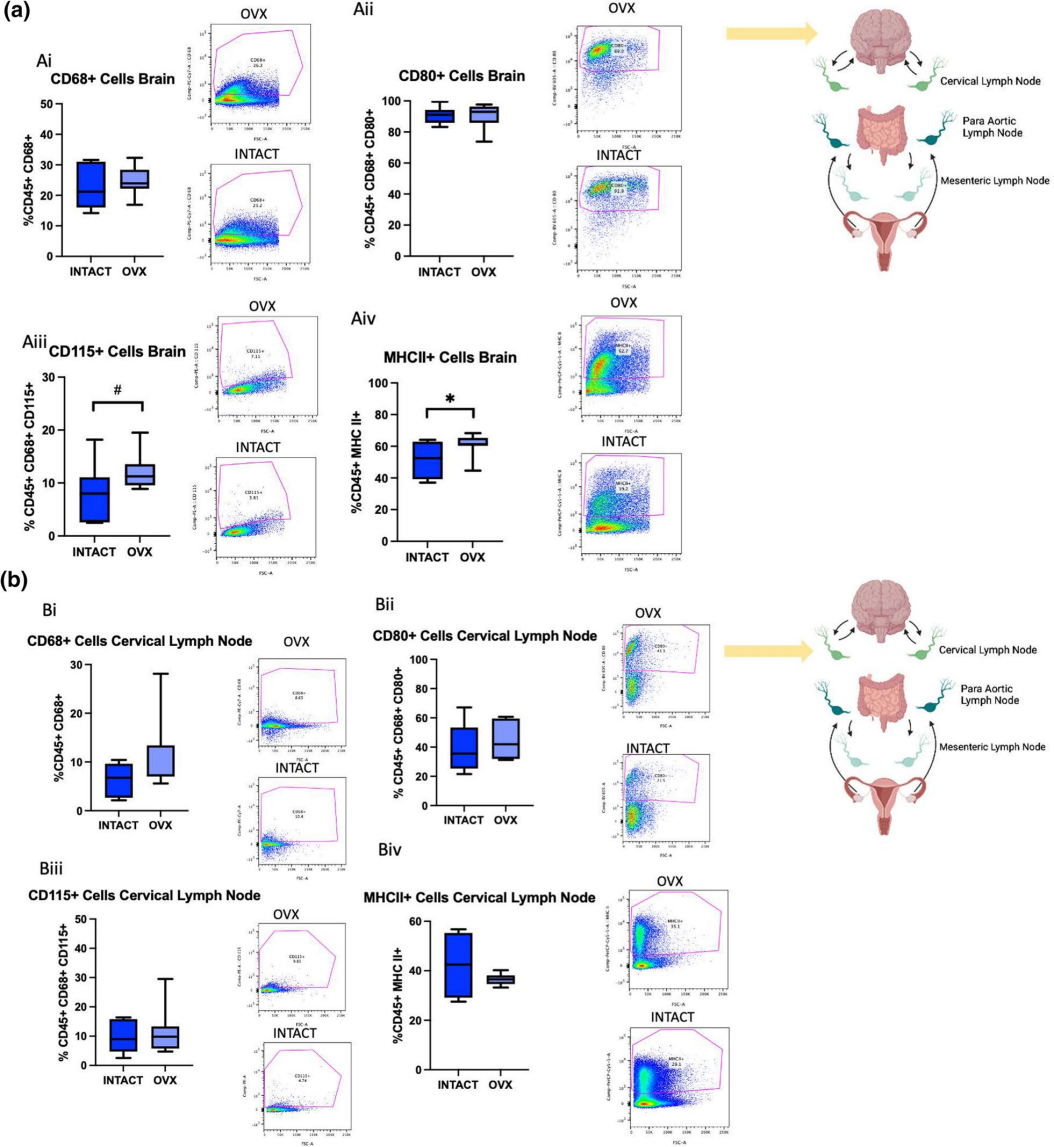

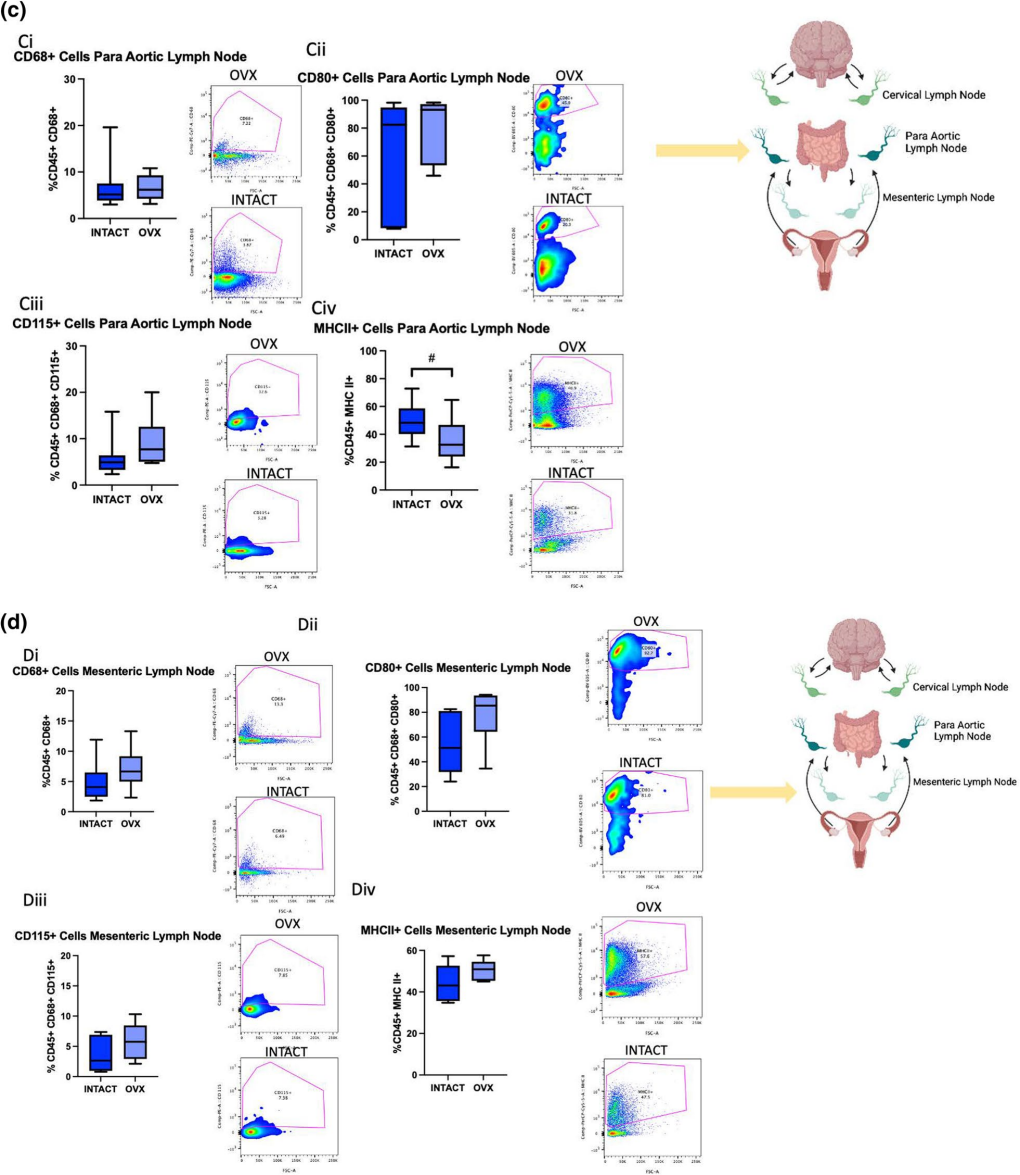

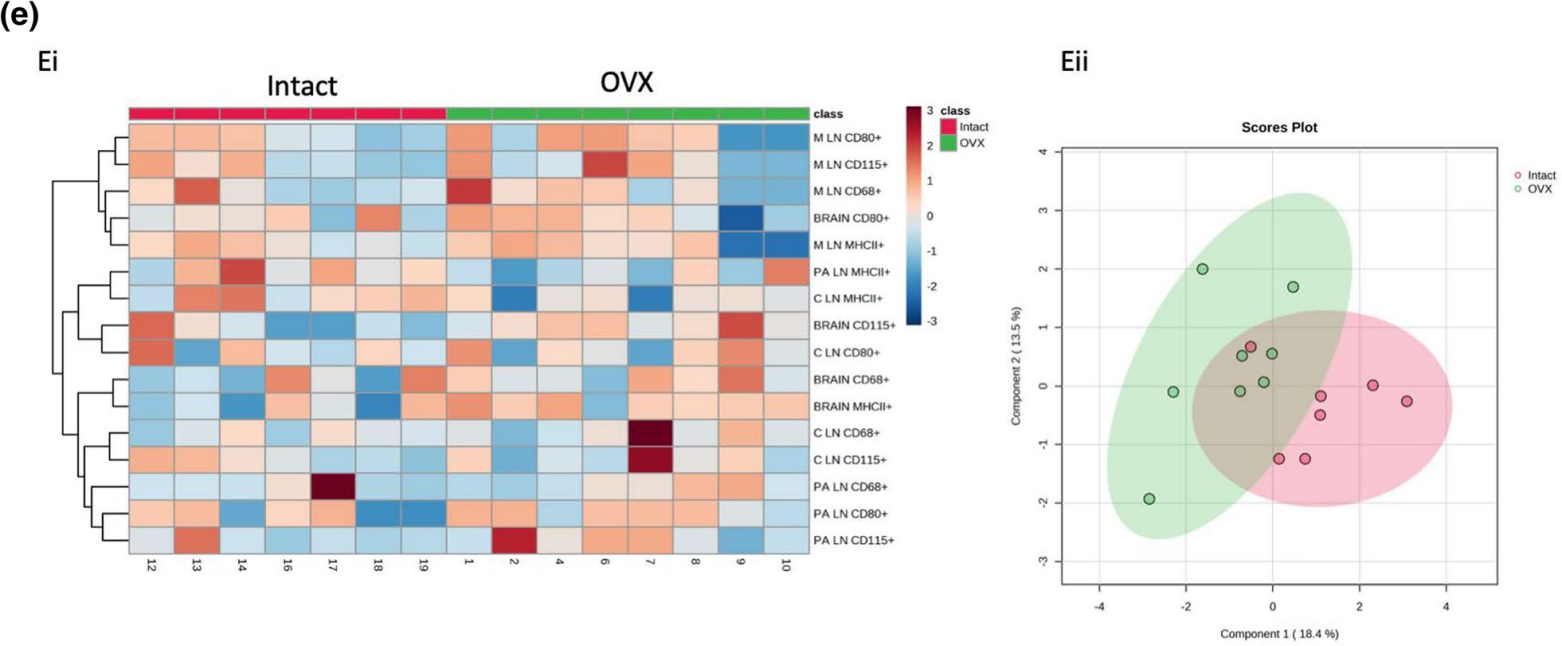
Macrophage flow cytometry analysis of brains and lymph nodes after ovariectomy.** A** Flow cytometry analysis of the brain of CD68 + (i) CD80 + (ii), CD115 + (iii), and MHCII + (iv) cells in RS intact and OVX females. **B** Flow cytometry analysis of the cervical lymph nodes of CD68 + (i) CD80 + (ii), CD115 + (iii), and MHCII + (iv) cells in RS intact and OVX females. **C** Flow cytometry analysis of the para-aortic lymph nodes of CD68 + (i) CD80 + (ii), CD115 + (iii), and MHCII + (iv) cells in RS intact and OVX females. **D** Flow cytometry analysis of the mesenteric lymph nodes of CD68 + (i) CD80 + (ii), CD115 + (iii), and MHCII + (iv) cells in RS intact and OVX females. **Ei** Heatmap analysis of immune cells populations in RS OVX and intact cervical, para-aortic, mesenteric lymph and brain post-stroke. **Eii** Sparse partial least squares discriminant analysis of macrophage populations in RS OVX and intact cervical, para-aortic, mesenteric lymph and brain post-stroke. All samples were collected 2 days post-stroke, and Student’s t test was used to compare groups. N = 7 (intact), N = 8 (OVX). *p ≤ 0.05

Brains from the OVX group showed no difference in overall macrophages (Fig. 7Ai) or in CD80 + macrophages (Fig. 7Aii). There was, however, a trend for an increase in CD115 + macrophages (Fig. 7Aiii, *p*= 0.0971) in the RS OVX group. Additionally, the OVX group indicates a significant increase in MHC II + cells (Fig. 7iv, *p*= 0.0540). In the cervical lymph nodes, there were no differences in any of the macrophage populations or MHC II + cells (Fig. 7Bi-iv). In the para-aortic lymph nodes, there were no difference in overall, M1, or M2 macrophages (Fig. 7Ci-iii). There was a trending reduction in MHCII + cells in the OVX group (Fig. 7Civ, *p*= 0.0891). There were no differences in macrophage populations in the mesenteric lymph nodes (Fig. 7Di-iv). To broadly assess the effect of ovariectomy on macrophage populations in these tissues in RS females post-stroke, data from this experiment visualized using a heatmap and a sparse partial least squares discriminant analysis (sPLS-DA) score (Fig. 7Ei-ii). The sPLS-DA scores plot shows populations that are orthogonal to each, indicating distinct differences between the two groups (Fig. 7Eii).

**Fig. 8:**
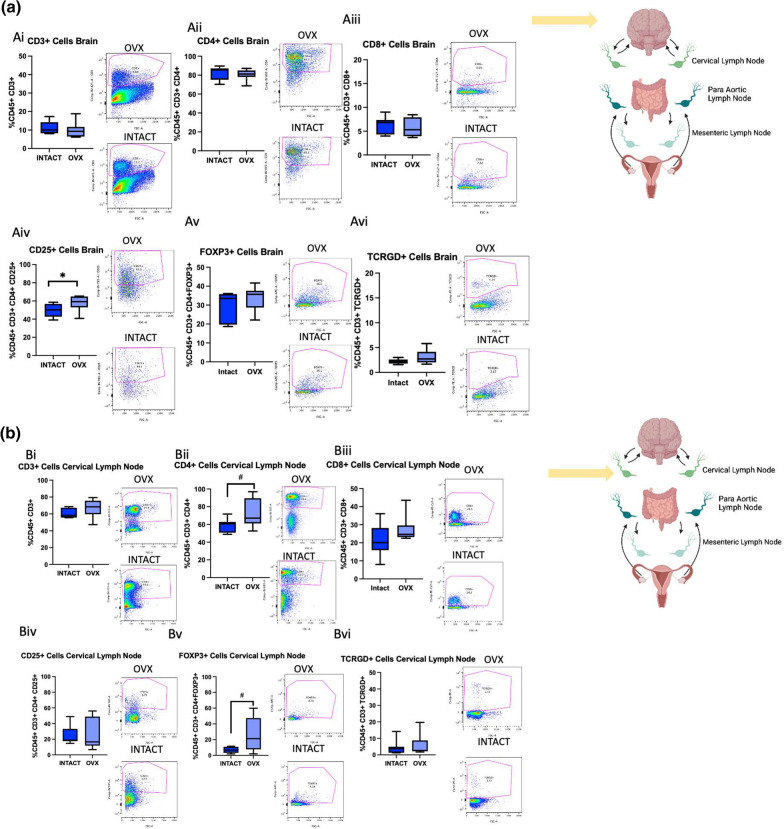

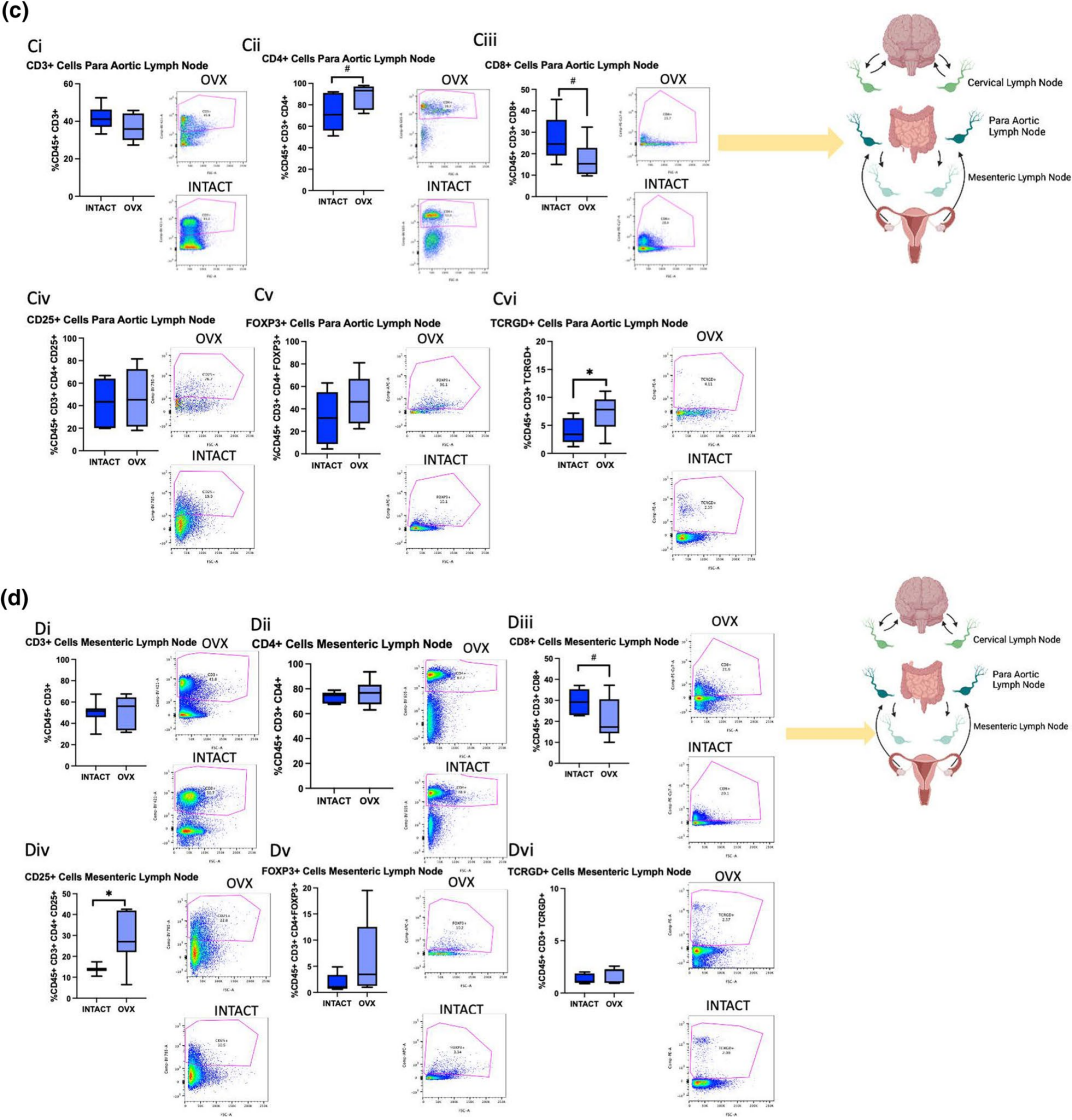

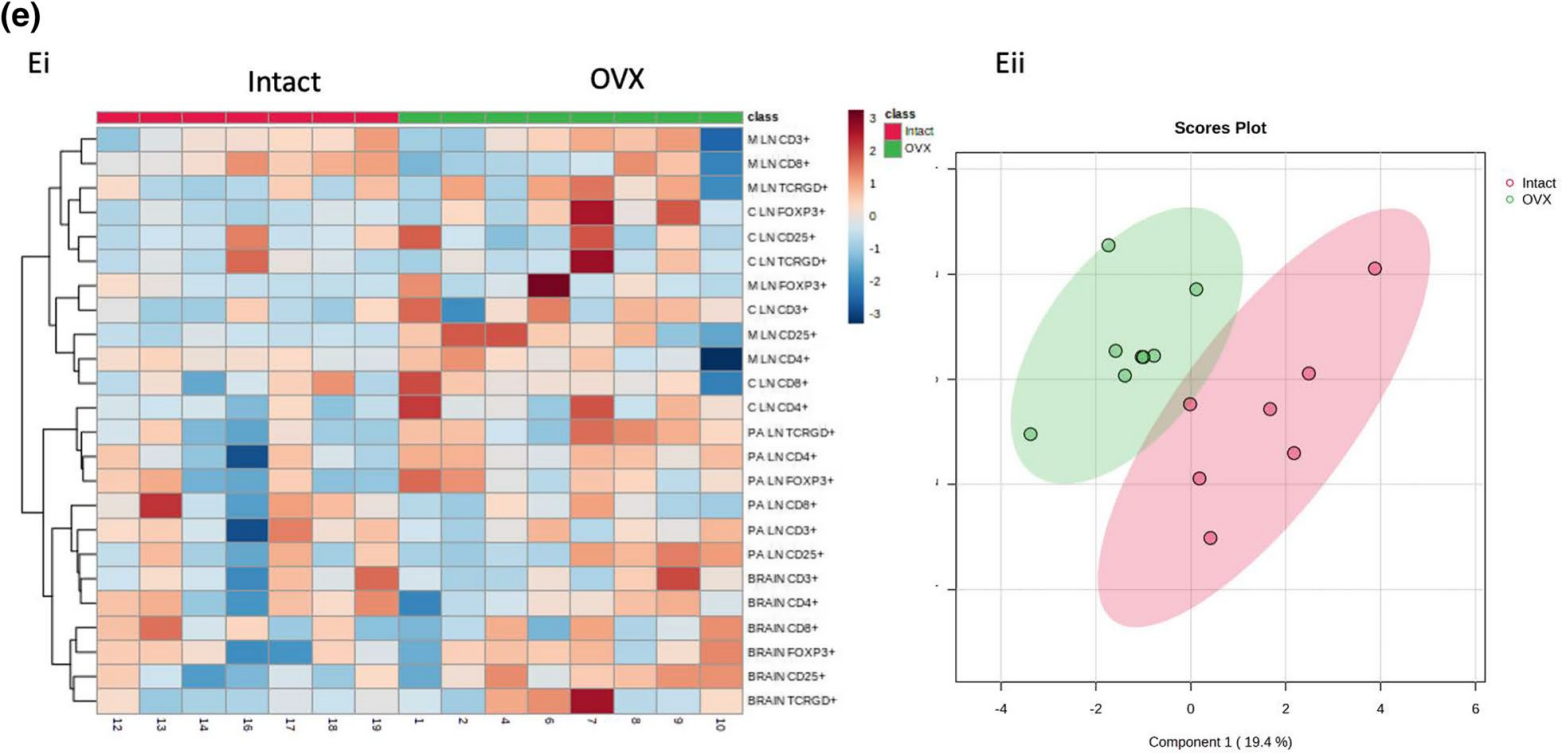
T cell flow cytometry analysis of brains and lymph nodes after ovariectomy. **A** Flow cytometry analysis of the brain of CD3 + (i) CD4 + (ii), CD8 + (iii), CD25 + (iv), FoxP3 + (v), and TCRGD + (vi) cells in RS intact and OVX females. **B** Flow cytometry analysis of the cervical lymph nodes of CD3 + (i) CD4 + (ii), CD8 + (iii), CD25 + (iv), FoxP3 + (v), and TCRGD + (vi) cells in RS intact and OVX females. **C** Flow cytometry analysis of the para-aortic lymph nodes of CD3 + (i) CD4 + (ii), CD8 + (iii), CD25 + (iv), FoxP3 + (v), and TCRGD + (vi) cells in RS intact and OVX females. **D** Flow cytometry analysis of the mesenteric lymph nodes of CD3 + (i) CD4 + (ii), CD8 + (iii), CD25 + (iv), FoxP3 + (v), and TCRGD + (vi) cells in RS intact and OVX females. **Eii** Sparse partial least squares discriminant analysis of macrophage populations in RS OVX and intact cervical, para-aortic, mesenteric lymph and brain post-stroke. All samples were collected 2 days post-stroke, and Student’s t test was used to compare groups. *N* = 7 (intact), *N* = 8 (OVX). *p ≤ 0.05

There were no differences in overall brain T cells, CD4 + T cells, or CD8 + T cells between the groups (Fig. 8Ai-Aiii). However, the OVX group showed increased brain CD25 + cells (Fig. 8Aiv, *p*= 0.0535), though there were no differences in FoxP3 + cells or $\gamma \delta$ T cells (Fig. 8Av-vi). For the cervical lymph nodes, there were trending increases in CD4 + T cells (*p* = 0.0693) and FoxP3 + T cells (* p*= 0.0597) in the OVX group, though there were no differences in any other T cell populations (Fig. 8Bi-vi). In the para-aortic lymph nodes, the OVX group demonstrated a trending increase in CD4 + T cells (Fig. 8Cii, *p*= 0.0589), a trending decrease in CD8 + T cells (Fig. 8Ciii, *p*= 0.0733), and a significant increase in $\gamma \delta$ T cells (*p*  = 0.0463), though no other differences in T cell populations were observed (Fig. 8Ci-vi). The only differences observed in T cell populations in the mesenteric lymph nodes was a trending decrease in CD8 + T cells (*p* = 0.0619) and a significant increase in CD25 + T cells in the OVX group (Fig. 8Di-vi, *p* = 0.0107). A heat map was created to visualize the differences in T cell populations in the OVX and intact groups (Fig. 8Ei). The sPLS-DA score for T cell populations of these groups show distinct separation, indicating robust differences in these cell populations (Fig. 8Eii).

## Discussion

The present study is the first to implicate the ovary as a key regulator of acute stroke outcomes with reproductive age playing a critical role in whether those outcomes are favorable or not. First, this study showed that simply removing the aging ovary prior to stroke resulted in reduced infarct volume and improved sensorimotor function. ELISA and LCMS analyses showed that the RS ovary and the Adult ovary respond differently to stroke with regard to cytokine production and hormone synthesis. Moreover, populations of immune cells are shown to be altered by stroke with a greater number of overall T cells being depleted and Tregs and M2-like macrophages being retained in the aging ovary post-stroke. Comparatively, RS OVX resulted in altered macrophage and T cell populations in the brain and lymph nodes that were analyzed, with the OVX group demonstrating an overall less inflammatory phenotype.

Stroke is primarily thought of as a brain-specific injury; however, recent studies, including this one, demonstrate that stroke has a profound effect on the entire body. In the most extreme cases of ischemic stroke, this injury can result in multi-system organ failure, including acute lung injury, liver failure, and kidney failure [31]. Previous studies from our lab have shown that stroke has a profound sex-specific impact on gut architecture (implicating sex hormones as a regulator of this phenomenon) [32] and that repairing the gut is capable of robustly improving stroke outcomes [33]. Other studies have shown that the gut traffics immune cells to the brain post-stroke [28] and that dysbiosis prohibits effector T cells from traveling to the meninges post-stroke [30]. This study introduces the ovary as another modulator of stroke outcome, potentially serving as an extra pool of immune cells that, when eliminated, results in less severe brain injury. This phenomenon is specific to the aging ovary, as ovariectomy of normally cycling Adult females exacerbates stroke damage [7], which implicates sex hormones as a regulator of immune cell populations in the ovary. That is, the presence of these sex hormones influences either the function of or populations of immune cells that exist within the ovary, which is consistent with previous studies as most immune cells express either ER $\alpha$ or ER $\beta$ [34]. Moreover, in postmenopausal women, monocytes show elevated expression of ER $\alpha$ as compared to premenopausal women, indicating that as estrogen levels decrease, ER $\alpha$ expression increases [35]. Our lab has also shown that RS rats show increased ER $\alpha$ expression in the forebrain as compared to adult females [16]. However, the effect of estrogen on immune function is quite complex. For example, estradiol treatment in in vitro cultures enhances B cell maturation by inhibiting T cell suppression of this phenomenon [36]. Estradiol has been shown to promote pathways involved in the production of type I IFN [37–39] and promote pro-inflammatory cytokine production in response to toll-like receptor ligand stimulation on dendritic cells and macrophages [40–42]. However, other studies have shown estradiol to have anti-inflammatory properties. For example, estradiol has been shown to inhibit NF-$\kappa$ B activity, resulting in a reduced inflammatory response [43]. Our data showing that the aging ovary, with a different hormonal profile than the adult ovary, has different immune cell populations and responds differently to stroke is consistent with the idea that estrogens profoundly alter immune function.

This study also shows a reduction in neural expression of VEGF and GM-CSF post-stroke in the OVX group. VEGF is known to increase in the brain post-stroke [44, 45]. While some studies have identified the angiogenic effects of VEGF to be neuroprotective, others have shown that endogenously upregulated or exogenously administered VEGF after stroke is detrimental, which could be due to VEGF-mediated blood–brain barrier breakdown and vascular leakage [46]. GM-CSF treatment, however, has been shown to be neuroprotective after MCAo in mice by reducing infarct size and enhancing leptomeningeal collateral growth [47]. In humans, plasma levels of GM-CSF are endogenously upregulated after stroke and were positively correlated with stroke severity score [48], which may indicate that the increase in this neuroprotective chemokine may be a compensatory mechanism.

An intriguing aspect of this study is that stroke has a profound effect on the ovarian synthesis of steroid hormones, with every hormone being downregulated post-stroke in both the adult and the RS ovaries. Though many studies have assessed the effects of these hormones on stroke outcomes, few have analyzed the converse. One clinical study showed no significant different in estradiol level in women 24 h post-stroke [49] (*p* = 0.10), although androgen levels decrease after ischemic stroke in men [49, 50]. However, a meta-analysis showed that women who experienced an adverse cardiovascular event before the age of 35 had twice the risk of early menopause (prior to 45), as compared to women who did not experience these early-life adverse cardiovascular events [51], indicating there could be a persistent effect of stroke on ovarian function. One possible reason that this phenomenon has not received much attention is because this premenopausal population experiences much fewer strokes as compared to postmenopausal women. However, trauma-induced disruption to the menstrual cycle is a well-known symptom of spinal cord injury and traumatic brain injury. One study reported 77.5% of normally cycling women experience amenorrhea after spinal cord injury [52]. Traumatic brain injury studies report a reduction in circulating estradiol in 43% of women in the days following injury [53], and 68% of women experiences irregular cycles for up to 60 months post-injury, with 46% of those women experiencing amenorrhea [54]. The findings in this paper that stroke results in a steep drop in ovarian hormone synthesis is therefore consistent with literature that suggests that neurotrauma significantly impairs ovarian hormone production and function. Though the current study did not reveal a difference in the ratio of ovarian estradiol to testosterone, we did observe a significant decrease in serum estradiol/testosterone in RS OVX animals as compared to the RS intact group. This is consistent with clinical data that greater serum estradiol/testosterone ratio is associated with an increased risk for stroke [55, 56].

Flow cytometry analysis of the ovaries in this study showed an unusually high proportion of CD4 + T cells and CD80 + macrophages and a relatively lower proportion of CD8 + T cells in the ovaries of all groups. Unfortunately, due to a paucity of data on the immune composition of the healthy and normally cycling rat ovary, much less the acyclic or stroke-injured ovary, it is difficult to speculate whether these results represent typical rat ovary lymphocyte or monocyte populations. The literature is clear, however, that ovariectomy does have a profound impact on immune cell populations of other organs, though many of these studies focus only on ovariectomy in reproductively competent animals. One study showed 20-month-old rats that were ovariectomized at the end of their reproductive period have a greater number of CD8 + blood lymphocytes and splenocytes as well as more CD4 + FoxP3 + blood lymphocytes and splenocytes [57]. A different study showed that ovariectomy at 1 month of age increased the CD4 + and CD8 + cell counts in the peripheral blood and spleen one month later. Moreover, the ovariectomized animals showed reduced apoptosis of these cells as compared to their intact counterparts [58]. In the ovaries of these RS rats, we see a significant reduction in the number of CD3 + cells, and an increase in the number of anti-inflammatory cells (CD115 + macrophages and CD25 + and FoxP3 + T cells). We propose that stroke depletes immune cells in the aging ovary with a bias towards extravasation of more inflammatory cells. However, the current study cannot exclude the possibility that these cells are instead dying in the ovary as we did not assess cell death of these populations. Interestingly, our study also indicates an abnormally high proportion of $\gamma \delta$ T cells in the ovaries. Little is known about ovarian $\gamma \delta$ T cells, though one study did show that co-culturing T cells with bovine luteal cells induced a $\gamma \delta$ T cell phenotype [59]. Therefore, it is plausible that luteal cells in the ovaries induces this high proportion of $\gamma \delta$ T cells.

Removal of the aging ovary significantly altered immune cell populations in the brain post-stroke. There was a trending increase in the number of CD115 + macrophages. This larger proportion of CD115 + cells in the brain may be beneficial for stroke outcome, as others have shown that CD115 + cells primed with anti-inflammatory chemokines injected into the cerebrospinal fluid post-stroke promote motor recovery and improve cognitive function [60]. The para-aortic and cervical lymph nodes also indicate a significant increase in the number of CD115 + macrophages in the OVX group. There was also an increase in MHCII + cells in the brains of the OVX group, which is likely neuroprotective. MHC Class II constructs have been shown to mitigate cell death in traumatic brain injury and stroke and are being investigated as potential therapeutics [61]. Lee et al. showed that treatment with an MHC Class II construct reduced stroke-induced motor deficits, neuroinflammation, infarct size, and cell death in the peri-infarct region[62]. Though the RS OVX group did not show a difference in overall CD3 + , CD4 + , or CD8 + T cells in the brain, they do show a significant increase in CD4 + CD25 + T cells. Liesz et al. suggested that these regulatory T cells likely protect the brain post-ischemia by preventing neuroinflammation, as anti-CD25 treatment prior to MCAo resulted in larger infarct size [63]. However, other studies have shown that Tregs exacerbate brain damage post-stroke [64]. Our study supports the notion that brain Tregs are beneficial for ischemic stroke, as the OVX group showed both higher populations of these cells and significantly better stroke outcomes. In the OVX animals, the para-aortic and mesenteric lymph nodes both indicate a trending reduction in CD8 + T cells. The cervical and para-aortic lymph nodes demonstrated increases in regulatory T cell populations; however, there is also a significant increase in $\gamma \delta$ T cells in the para-aortic lymph nodes. Overall, analysis of the immune cell populations of the lymph nodes generally indicates a less inflammatory in the OVX group as compared to the intact group.

This study shows that ovariectomy in reproductively senescent females results in better stroke outcomes, and our data suggest that this phenomenon is cannot be attributed solely to hormone synthesis, but rather that the aging ovary has a profound impact on neural and peripheral inflammation post-stroke. This study suggests the aging ovary is more than just a dormant organ that loses function post-menopause, but it instead actively and critically contributes to acute stroke outcomes and likely many other injury models that involve a reactive immune response, calling for more investigation in to how the aging ovary contributes to women’s health.

## Supplementary Information

**Additional file 1: Figure S1.** Effect of ovariectomy on body weight. **A** Histogram depicting meanof body weight pre-OVX, pre-MCAo, and 5d post-MCAo. **B** Histogram depicting meanof weight lost between MCAo and termination. **C** Histogram depicting meanof spleen weight normalized to body weight at termination. *N* = 14and *n* = 14. **p ≤ 0.01. **Figure S2.** Effect of age, ovariectomy, and stroke on serum cytokine expression. Multiplexed cytokine analysis of EGF, MCP-1, MIP-2, IL-1 $\alpha$in Adult and RS OVX and intact serum pre and 2 days post-MCAo. *N* = 5, *N* = 4, *N* = 5, and *N* = 5. **a** effect of stroke, **b** effect of age, **c** effect of OVX. *p ≤ 0.05. **Figure S3.** Effect of ovariectomy on serum steroid hormone expression. LC–MS analysis shows expression of estrone, testosterone, estradiol, and the ratio of estradiol/testosterone **D** in the serum of post-stroke Adult and RS OVX and intact. *N* = 6, *N* = 7, *N* = 5, *N* = 10. **a** effect of OVX, **b** effect of age, **c** interaction effect. *p ≤ 0.05. **Figure S4.** Effect of stroke on serum cytokine expression. Multiplexed cytokine analysis of IL-1 $\beta$, IL-17 $\alpha$, Eotaxin, IL-4, MCP-1, G-CSF, TNF- $\alpha$, Fractalkine, IL-5, IL12p70, Rantesand IL-2of Adult and RS sham and stroke serum. *N* = 10, *N* = 10, *N* = 7, *N* = 5. **a** Main effect of stroke; **b** main effect of age; **c** interaction of stroke by age. *p ≤ 0.05. **Figure S5. **Gating Strategy for flow cytometry experiments. Cells were gated based on side scatter area versus forward scatter area. Single cells were gated based on forward scatter height versus forward scatter area. Live cells were stained using GHOST-780 Live/Dead dye and gated using APC-Cy7 area versus forward scatter area. CD45 + cells were stained using a CD45-FITC antibodyand gated using FITC area versus forward scatter area. All data area reported as percentage of events.

## Data Availability

All data generated or analyzed in this study are included in the manuscript.

## Abbreviations

RS: Reproductively senescent
OVX: Ovariectomized
MCAo: Middle cerebral artery occlusion
Treg: Regulatory T cell
PCOS: Polycystic ovarian syndrome
ET-1: Endothelin-1
ART: Adhesive Removal Task
Vib: Vibrissae-evoked forelimb placement task
ELISA: Enzyme-linked immunosorbent assay
LC–MS: Liquid chromatography–mass spectrometry
IL: Interleukin
IFN$\gamma$: Interferon gamma
RANTES: Regulated upon Activation, Normally T cell Expressed and presumably Secreted
MCP-1: Monocyte chemoattractant protein 1
TNF-$\alpha$: Tumor necrosis factor alpha
G-CSF: Granulocyte colony-stimulating factor
CD: Cluster of differentiation
MHC II: Major histocompatibility complex class 2
SEM: Standard error of mean
sPLS-DA: Sparse partial least squares discriminant analysis
