# Supplementary figures and images for: The aging ovary impairs acute stroke outcomes
Source: J Neuroinflammation. 2023 Jul 5;20:159. doi: 10.1186/s12974-023-02839-1 (PMC10320896; doi:10.1186/s12974-023-02839-1)

# Additional File 1

Figure S1

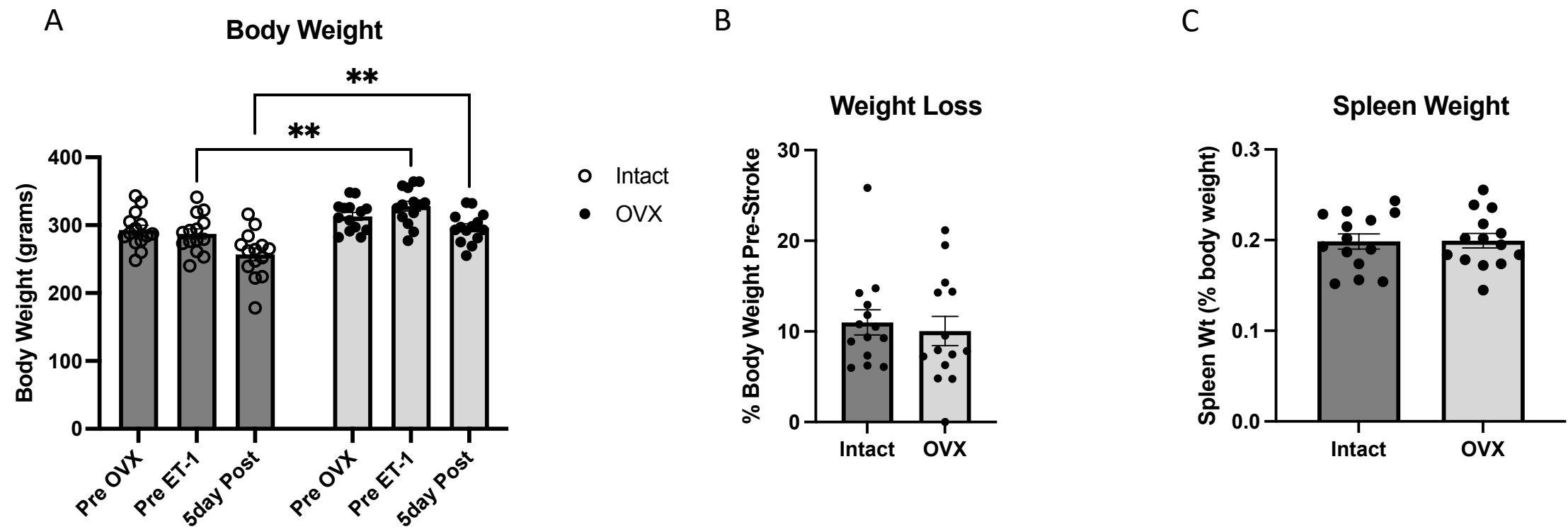

Figure S2

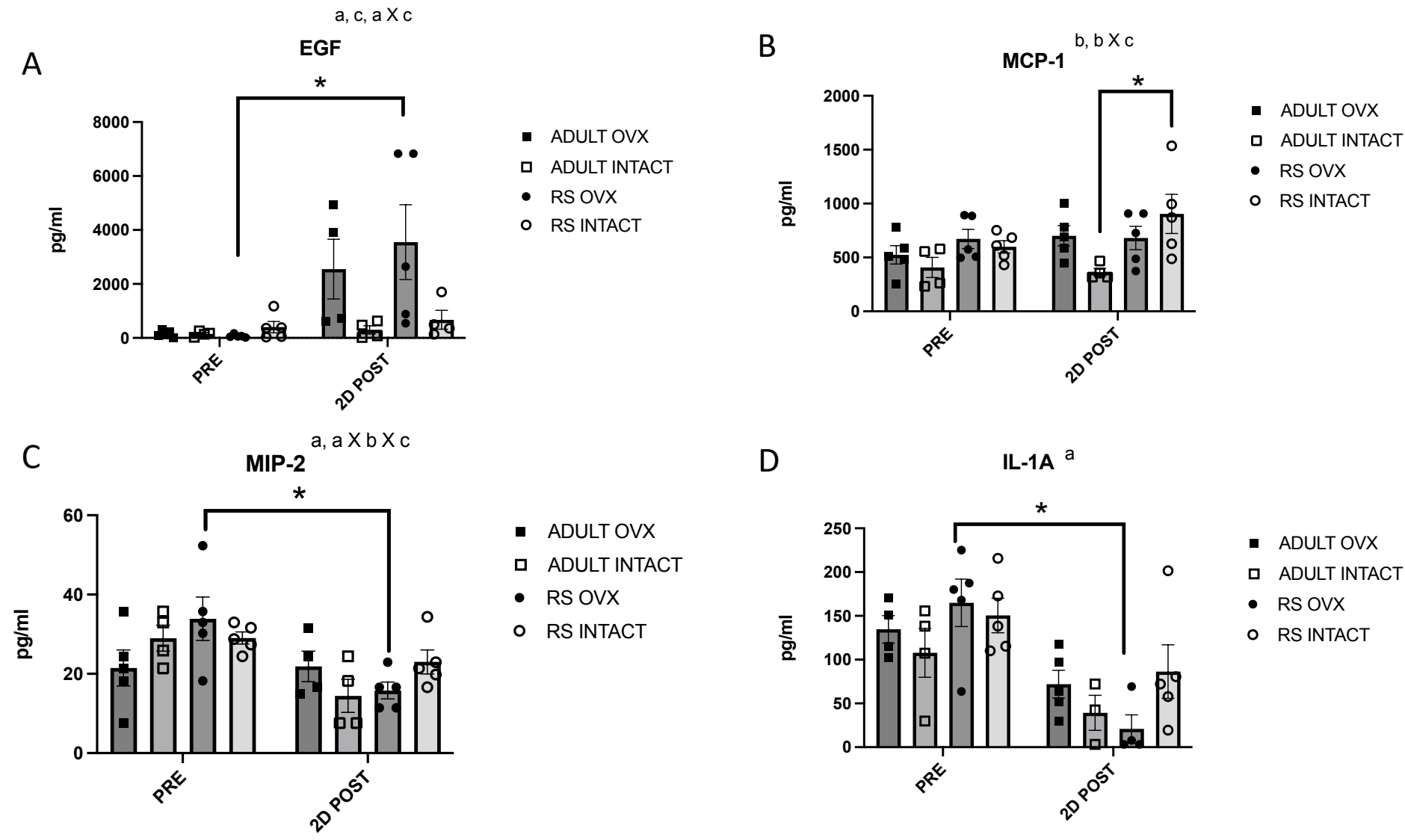

Figure S3

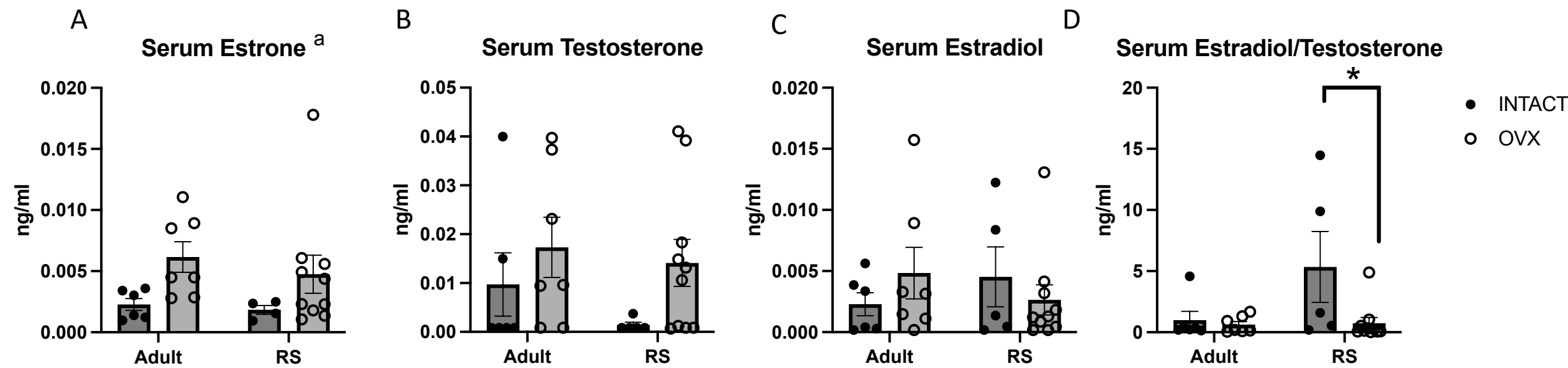

Figure S4

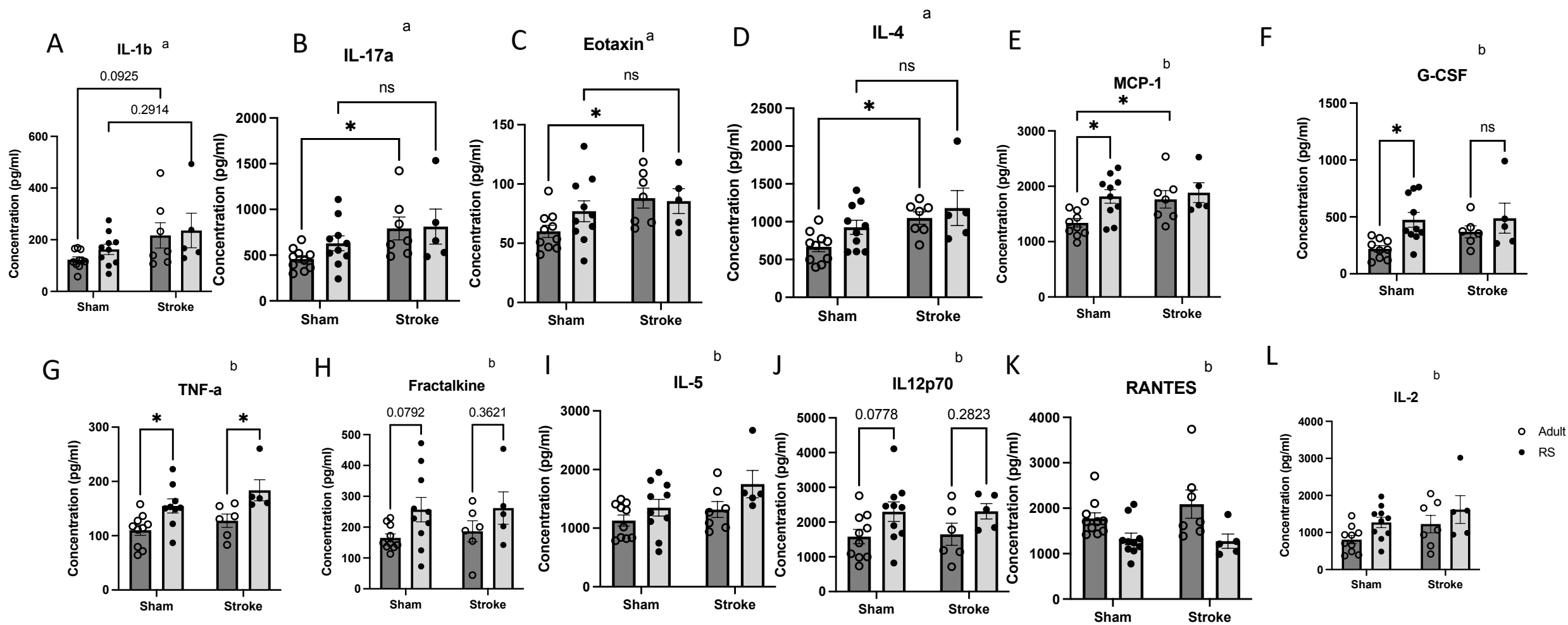

Figure S5

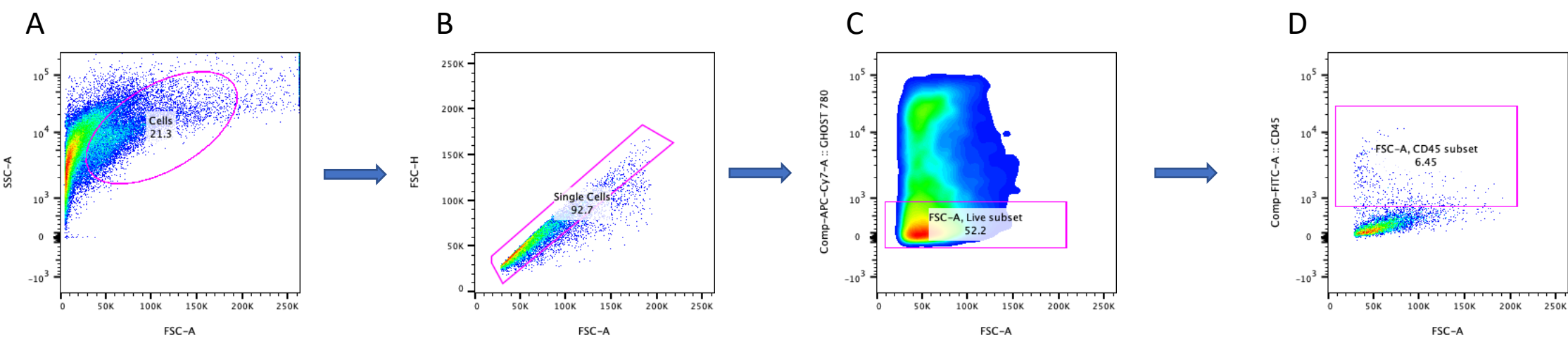

Supplement: Supplementary file 1 — Additional file 1: Figure S1. Effect of ovariectomy on body weight. A Histogram depicting meanof body weight pre-OVX, pre-MCAo, and 5d post-MCAo. B Histogram depicting meanof weight lost between MCAo and termination. C Histogram depicting meanof spleen weight normalized to body weight at termination. N = 14and n = 14. **p ≤ 0.01. Figure S2. Effect of age, ovariectomy, and stroke on serum cytokine expression. Multiplexed cytokine analysis of EGF, MCP-1, MIP-2, IL-1 $\alpha$αin Adult and RS OVX and intact serum pre and 2 days post-MCAo. N = 5, N = 4, N = 5, and N = 5. a effect of stroke, b effect of age, c effect of OVX. *p ≤ 0.05. Figure S3. Effect of ovariectomy on serum steroid hormone expression. LC–MS analysis shows expression of estrone, testosterone, estradiol, and the ratio of estradiol/testosterone D in the serum of post-stroke Adult and RS OVX and intact. N = 6, N = 7, N = 5, N = 10. a effect of OVX, b effect of age, c interaction effect. *p ≤ 0.05. Figure S4. Effect of stroke on serum cytokine expression. Multiplexed cytokine analysis of IL-1 $\beta$β, IL-17 $\alpha$α, Eotaxin, IL-4, MCP-1, G-CSF, TNF- [file 12974_2023_2839_MOESM1_ESM.pdf]
